# Clinical effects of omega-3 fatty acids supplementation in the periodontal treatment of smokers and non-smokers with periodontitis: a retrospective study

**DOI:** 10.1007/s00784-024-05835-8

**Published:** 2024-07-20

**Authors:** Levent Savran, Mehmet Sağlam

**Affiliations:** Department of Periodontology, Faculty of Dentistry, Izmir Katip Çelebi University, Izmir, Turkey

**Keywords:** Periodontitis, Non‑surgical treatment, Omega-3, Smoking

## Abstract

**Objectives:**

Omega-3 supplementation as an adjunct to nonsurgical periodontal treatment has been reported to have a positive effect on healing in periodontitis patients. However, there is a lack of information on the effects of periodontal healing in smokers with periodontitis. The aim of this retrospective study was to investigate the effect of omega-3 supplementation given as an adjunct to nonsurgical periodontal treatment on clinical parameters in smoker and non-smoker periodontitis patients.

**Methods:**

This study included a total of 80 periodontitis patients, 40 non-smokers and 40 smokers who were systemically healthy. In this study, patients were divided into 4 groups as follows: Group 1 (Subgingival instrumentation (SI) alone/nonsmoker), Group 2 (SI alone/smoker), Group 3 (SI + Omega-3/nonsmoker) and Group 4 (SI + Omega-3/smoker). Group 3 and 4 consumed 1320 mg Omega-3 capsule (640 mg EPA, 480 mg DHA) once a day for 3 months. Probing depth (PD), clinical attachment level (CAL), gingival index (GI), plaque index (PI) and bleeding on probing (BOP %) were recorded at baseline, 1 month and 3 months after treatment.

**Results:**

Significant improvement of all clinical parameters at 1 and 3 months was observed in all groups. Whole mouth CAL, GI and BOP% were significantly reduced in group 4 compared to group 2 at 1 and 3 months postoperatively (*p* < 0.05). For moderately deep pockets (4–6 mm) and deep pockets (7 mm≤), PD and CAL reductions were significantly greater in groups taking omega − 3 (group 3 and group 4) compared to groups not taking omega-3 (group 1 and group 2) between baseline and 1 month and between baseline and 3 months (p ˂ 0.05).

**Conclusion:**

Omega-3 supplementation given as an adjunct to nonsurgical periodontal treatment provided significant benefit in the improvement of clinical parameters (especially for CAL and PD) in the short term in smokers and non-smokers with periodontitis.

**Clinical relevance:**

Nonsurgical periodontal treatment with omega-3 supplementation resulted in significant improvements in clinical parameters in smokers and non-smokers with periodontitis.

## Introduction

The onset of periodontal disease is triggered by the existence and buildup of bacterial pathogens. However, the advancement of the disease is linked to the transition of the host-microorganism symbiotic relationship into a pathogenic dysbiotic relationship [1]. Periodontal disease starts as inflammation called gingivitis, which involves only the gingiva and can progress to periodontitis, characterized by the destruction of tooth-supporting tissues in susceptible individuals [2]. Although periodontal disease is initiated by bacterial biofilm, host response is the most important factor in periodontal tissue destruction and response to treatment [3]. Susceptibility to periodontitis and the progression of periodontitis are influenced by a variety of variables. A person’s behavior, such as smoking and oral hygiene habits, genetic factors, systemic disease like diabetes, and genetic factors, are among these.

Smoking poses a significant risk for developing periodontitis and may also affect the progression of periodontal disease. Smokers are more likely to have severe periodontal disease, which is characterized by increased probing depth, clinical attachment loss, gingival recession substantial bone loss, and a high risk of tooth loss [4]. Additionally, non-surgical periodontal therapy is negatively affected by smoking [5].

Subgingival instrumentation (SI) combined with efficient supragingival biofilm management is considered the gold-standard treatment for periodontitis [6, 7]. Despite the well-established effectiveness of non-surgical periodontal therapy, there are some drawbacks that lower its success rate, such as tissue-invading microorganisms and inaccessible regions like deep pockets, concavities and grooves on the root surfaces, furcation involvement, and the distal sites of molars [8]. The main causes of periodontal therapy failure are an inability to correct biofilm dysbiosis and manage inflammation [9]. Antimicrobial medicines and immune modulators have been considered to treat periodontitis.

Recently, increased focus has been placed on omega-3 polyunsaturated fatty acids (PUFAs), such as eicosapentaenoic acid (EPA) and docosahexaenoic acid (DHA), as a host modulator. Utilizing omega-3 fatty acids prevents the production of pro-inflammatory molecules like cytokines and eicosanoids [10]. Omega-3 fatty acids can be converted into resolvin D, resolvin E, protectins, and neuroprotectins. These compounds, known as specialized pro-resolving mediators (SPMs), play a crucial role in controlling and resolving inflammation [11]. EPA and DHA have the ability to create specific SPMs. Resolvin D series, protectins, and maresins are produced by DHA, while resolvin E series are produced by EPA [12, 13]. These SPMs are pro-resolving molecules, which reduces tissue damage and improves tissue protection during acute inflammation [14]. Resolvins and protectins also have antioxidative properties [15]. Patients with various diseases, including ulcerative colitis, cardiovascular disorders, asthma, rheumatoid arthritis, and periodontitis, were found to benefit from the therapeutic, anti-inflammatory, and preventive effects of omega-3 PUFAs [16].

Van Dyke et al. stated that SPMs would support periodontal healing and even periodontal regeneration by activating the pro-resolving circuit [17]. EPA and DHA have been found to have inhibitory effects on bacteria in addition to their anti-inflammatory and antioxidant benefits. Inhibiting the action of periodontal pathogens such as *Aggregatibacter actinomycetemcomitans*, *Porphyromonas gingivalis*, *Prevotella intermedia*, *Tannerella forsythia*, *Treponema denticola*, and *Fusobacterium nucleatum* was shown to be possible in several studies [10, 18, 19]. Additionally, it has been claimed that omega-3 PUFAs inhibit enzymes like matrix metalloproteinase (MMP)-2 and MMP-9, which are involved in the destruction of periodontal tissue [20]. In the literature, there are various studies examining the effect of omega-3 supplementation on clinical, biochemical, and microbiological parameters in addition to non-surgical periodontal treatment. According to these studies, taking omega-3 PUFAs improved clinical measures such as probing depth (PD), clinical attachment level (CAL), gingival index (GI), and bleeding on probing (BOP) [21–27]. From a biochemical point of view, it has been reported that interleukin (IL)-1β, tumor necrosis factor (TNF)-α, IL-8, IL-17, RANKL, MMP-8, and pentraxin levels were significantly reduced in omega-3 PUFAs + SI groups compared to SI alone [23, 25, 28, 29]. In a recent study, Stando-Retecka et al. demonstrated that high‑dose omega‑3 PUFA intake significantly reduced *Aggregatibacter actinomycetemcomitans*,* Porphyromonas gingivalis*, *Tannerella forsythia*, and *Treponema denticola counts* at 6 months after non-surgical periodontal therapy compared to the control group [19].

Recent systematic reviews and meta-analyses have reported that the use of omega-3 PUFAs in addition to non-surgical periodontal therapy provides significantly better improvement, especially in PD and CAL parameters [30–34]. However, there is no study investigating the effect of omega-3 PUFAs use in addition to non-surgical periodontal therapy in smokers who have a reduced response to periodontal therapy. Periodontal healing may be more challenging to achieve when treating individuals with risk factors like diabetes or smoking particularly in deep pockets and on multirooted teeth. The additional use of omega-3 in those circumstances may be even more beneficial. In this retrospective study, it is hypothesized that omega-3 could be beneficial for the treatment of smokers and non-smokers with periodontitis. To test this hypothesis, the effects of omega-3 use on clinical parameters in the treatment of smokers and nonsmokers with periodontitis were evaluated retrospectively using clinical charts.

## Materials and methods

In this retrospective study, the data have been obtained from clinical studies conducted by Prof. Dr. Mehmet Sağlam and from the clinical records of patients whose treatments he has been monitoring. The retrospective collection of data was carried out after obtaining approval from the Izmir Katip Çelebi University Non-Interventional Clinical Research Ethics Committee (No: 0028/2023). The principles specified in the 1975 Helsinki Declaration, updated in 2013, were followed in the conduct of this investigation. Written informed consent was obtained from each patient.

Both smokers (≥ 10 cigarettes a day) and nonsmokers (never smokers) with periodontitis, who had at least 20 teeth excluding 3rd molars in their mouths were included in the study. Radiographic bone loss with a PD of ≥ 5 mm and CAL of ≥ 4 mm in at least 2 teeth in each quadrant was selected as periodontitis criteria. For the omega-3 PUFAs groups, individuals who were recommended to use omega-3 supplements during the non-surgical treatment were included. Exclusion criteria were: (1) Having received periodontal therapy within the previous six months; (2) using steroid, antibiotic, or anti-inflammatory medication during the last six months; and (3) having any systemic illness, pregnancy, or breastfeeding and taking any medication.

Study groups were as follows:

- Group 1: nonsmoker SI alone (control 1 group) (*n* = 20).
- Group 2: smoker SI alone (control 2 group) (*n* = 20).
- Group 3: nonsmoker SI + Omega-3 PUFAs (test 1 group) (*n* = 20).
- Group 4: smoker SI + Omega-3 PUFAs (test 2 group) (*n* = 20).

## Clinical measurements

At baseline, 1 and 3 months after therapy, six sites (mesiobuccal, midbuccal, disto-buccal, mesiolingual/mesio-palatal, midlingual/mid-palatal, and disto-lingual/disto-palatal) were used to measure PD and CAL using a periodontal probe (Hu-Friedy, Chicago, IL, USA). Recordings were also made for GI, plaque index (PI), and BOP (%) at four sites (mesial, buccal, distal, and lingual/palatal). All clinical measurements were performed by periodontists.

## Procedures performed on patients

### Supragingival scaling and oral hygiene instructions

All patients underwent supragingival plaque and calculus debridement with hand instruments (Hu-Friedy, Chicago, IL, USA) and ultrasonic scalers (EMS Mini-Piezon, Nyon, Switzerland). All teeth were polished with a rubber cup. In the same session, all patients received detailed oral hygiene instructions including tooth brushing and interface cleaning. As part of the phase I periodontal treatment, the patients’ hopeless teeth were extracted and restorative treatment of the teeth with carious lesions was performed.

### SI

Each patient in all groups underwent full-mouth SI under local anesthetic one week following the supragingival scaling, utilizing Gracey curettes (Gracey Curets, Hu-Friedy, Chicago, IL, USA) and ultrasonic scalers. SI was performed until a smooth surface was felt for each periodontal pocket of 4 mm or more. Subgingival saline irrigation was performed adjunctively to SI. Following this treatment, patients were contacted for follow-up appointments one week, one month, and three months later. Oral hygiene motivation was repeated at the follow-up sessions. Clinical measurements were repeated at the 1st and 3rd month after SI by periodontists. Subgingival reinstrumentation was performed at BOP-positive sites with PD ≥ 4 mm and at all sites exhibiting PD ≥ 5 mm at 1 month and 3 months after SI.

### Additional omega-3 PUFAs supplementation

Omega-3 PUFAs supplement (1320 mg Omega-3 fatty acids, 640 mg EPA, 480 mg DHA, once a day for 3 months) (Möller’s, Oslo, Norway) as an adjunct to SI was given to all patients in test groups. Patients started taking Omega-3 supplements on the day of SI procedure. During the course of the treatment, the patients’ compliance with taking omega-3 capsules was monitored by calling them every four weeks to check the bottles for any possible remaining capsules and by asking information about their supplement use on a weekly basis.

### Sample size calculation and statistical analyses

The G*Power program, version 3.1.5 (Franz Faul, Christian-Albrechts Universität Kiel, Kiel, Germany), was used to calculate sample size. Sample size was determined using the CAL parameter, taking into account the previously published study [21]. According to power analysis software, this study required a minimum of 20 participants for each group with a power of 80% and α = 0.05. The statistical analysis was done using a software program (SPSS v. 29.0; IBM, Chicago, IL). The normality of the data was evaluated using the Kolmogorov-Smirnov test. Intergroup analysis for full-mouth clinical parameters involved the use of one-way analysis of variance (ANOVA) followed by Tukey’s test, while intragroup analysis was conducted using a repeated measures ANOVA test. Intergroup analysis for PD and CAL changes in moderate deep pockets and deep pockets involved the application of the Kruskal–Wallis test followed by Dunn’s test. Multiple regression analysis was performed to examine the influence of the independent variables (age, gender, smoking and omega-3 usage) on PD and CAL changes from baseline to 3 months for moderately deep pockets and deep pockets. *P* < 0.05 was considered statistically significant.

## Results

Table 1 presents baseline demographic data of all participants in study groups. There was not statistical difference between groups in terms of number of teeth, age and sex (*p* > 0.05).

**Table 1:** Demographic characteristics of the study groups

Study Groups	*N*	Number of teeth (Mean ± SD)		Age (Mean ± SD)		Sex		Cigarette consumption/day (Mean±SD)	Pack-year (Mean ± SD)	Moderate deeppockets (4–6 mm) percent and number	Deep pockets (7 mm≤) percent and number
						Male	Female				
Group 1	20	25.60	± 1.66	44.9	± 8.09	10	10	0±0a	0±0a	12% (367)	4% (151)
Group 2	20	24.75	± 2.17	44.7	± 9.24	11	9	19.75±4.43b	16.32±7.52b	17% (502)	2% (53)
Group 3	20	26.20	± 1.88	39.2	± 9.01	11	9	0±0a	0±0a	12% (374)	2% (69)
Group 4	20	25.35	± 2.99	39.8	± 10.32	9	11	18.50±5.40b	15.18±6.35b	16% (479)	2% (63)
p value		0.237		0.092		0.908		*p*<0.001	*p*<0.001		

Different letters indicate statistically significant difference for intergroup analyses

According to the groups, the severity and degree of progression of periodontitis were as follows:

- **Group 1**: 2 patients with Stage III Grade B periodontitis, 18 patients with Stage III Grade C periodontitis.
- **Group 2**: 20 patients with Stage III Grade C periodontitis.
- **Group 3**: 15 patients with Stage III Grade B periodontitis and 5 patients with Stage III Grade C periodontitis.
- **Group 4**: 17 patients with Stage III Grade C and 3 patients with Stage IV Grade C.

Whole mouth clinical parameters were presented in Table 2. All clinical parameters were reduced at 1 and 3 months after periodontal therapy compared to baseline in all groups (*p* < 0.001). Whole mouth PD was lower at 3 months compared to 1 month for all groups (*p* < 0.05). Only for group 4, whole mouth CAL, GI and BOP (%) was lower at 3 months compared to 1 month (*p* < 0.05). There was not significant difference between all groups at baseline in terms of whole mouth clinical parameters (*p* ≥ 0.05). At 1 and 3 months after therapy, whole mouth PD were lower in group 1 and group 3 compared to group 2 (*p* < 0.05). Whole mouth PD were similar between group 1, group 3 and group 4 (*p* > 0.05) and, also between group 2 and group 4 (*p* > 0.05) at 1 and 3 months postoperatively. Whole mouth CAL, GI and BOP (%) were significantly lower in group 1, group 3 and group 4 compared to group 2 (*p* < 0.05) at 1 and 3 months postoperatively. These clinical parameters were similar between group 1, group 3 and group 4 (*p* > 0.05). There was no significant difference between study groups in terms of PI (*p* > 0.05).

**Table 2:** Whole mouth clinical parameters (Mean ± SD)

PD (mm)	Baseline	1 month	3 months	*p*¶ value
Group 1	3.15 ± 0.49	2.28 ± 0.38a *	2.26 ± 0.39a *.†	*p* < 0.001
Group 2	3.51 ± 0.61	2.76 ± 0.48b *	2.73 ± 0.49b *.†	*p* < 0.001
Group 3	3.43 ± 0.71	2.28 ± 0.56a *	2.25 ± 0.54a *.†	*p* < 0.001
Group 4	3.18 ± 0.52	2.58 ± 0.50a.b *	2.54 ± 0.50a.b *.†	*p* < 0.001
p# value	0.144	0.005	0.005	

CAL (mm)				
Group 1	3.62 ± 1.10	2.51 ± 1.06a *	2.54 ± 1.08a *	*p* < 0.001
Group 2	4.33 ± 1.33	3.45 ± 1.06b *	3.41 ± 1.04b *	*p* < 0.001
Group 3	3.60 ± 0.78	2.30 ± 0.60a *	2.29 ± 0.59a *	*p* < 0.001
Group 4	3.57 ± 0.49	2.55 ± 0.47a *	2.53 ± 0.47a *.†	*p* < 0.001
p# value	0.05	*p* < 0.001	*p* < 0.001	

GI				
Group 1	1.63 ± 0.53	0.72 ± 0.36a *	0.65 ± 0.39a *	*p* < 0.001
Group 2	1.66 ± 0.14	1.05 ± 0.30b *	1.08 ± 0.46b *	*p* < 0.001
Group 3	1.82 ± 0.37	0.62 ± 0.19a *	0.64 ± 0.14a *	*p* < 0.001
Group 4	1.61 ± 0.21	0.80 ± 0.09a *	0.6 ± 0.08a *.†	*p* < 0.001
p# value	0.344	*p* < 0.001	*p* < 0.001	

PI				
Group 1	1.57 ± 0.44	0.74 ± 0.29 *	0.76 ± 0.31 *	*p* < 0.001
Group 2	1.79 ± 0.61	0.94 ± 0.40 *	0.97 ± 0.32 *	*p* < 0.001
Group 3	1.82 ± 0.51	0.82 ± 0.09 *	0.79 ± 0.13 *	*p* < 0.001
Group 4	1.60 ± 0.26	0.86 ± 0.07 *	0.86 ± 0.09 *	*p* < 0.001
p# value	0.249	0.108	0.06	

BOP (%)				
Group 1	51.98 ± 25.57	19.37 ± 8.32a *	16.22 ± 9.89a *	*p* < 0.001
Group 2	65.81 ± 22.50	31.07 ± 18.74b *	28.68 ± 14.64b *	*p* < 0.001
Group 3	65.53 ± 21.08	17.76 ± 4.68a *	15.48 ± 2.55a *	*p* < 0.001
Group 4	58.63 ± 19.18	22.23 ± 2.79a *	16.64 ± 7.20a *.†	*p* < 0.001
p# value	0.164	*p* < 0.001	*p* < 0.001	

p# value for intergroup analysis (ANOVA and Tukey test). Different letters indicate statistically significant difference for intergroup analyses

p¶ value for intragroup analysis (ANOVA for repeated measurements). *: Significant statistical difference from baseline. †: Significant statistical difference compared to the 1st month

PD and CAL changes for moderately deep pockets (4–6 mm) were shown in Table 3. PD reduction was greater in group 3 compared to other groups between baseline and 1 month and, between baseline and 3 months (*p* < 0.05). PD reduction was greater in group 1 compared to group 2 and group 4 (*p* < 0.05), and also greater in group 4 compared to group 2 between baseline and 1 month and, between baseline and 3 months (*p* < 0.05). CAL reduction was greater in group 3 compared to other groups between baseline and 1 month and, between baseline and 3 months (*p* < 0.05). CAL reduction was greater in group 1 compared to group 2 and group 4 (*p* < 0.05), and also greater in group 4 compared to group 2 between baseline and 1 month and, between baseline and 3 months (*p* < 0.05).

**Table 3:** PD and CAL changes (Mean ± SD/Median (Min.-Max.) For moderately deep pockets (4–6 mm)

Moderately deep pockets (4–6 mm) PD and CAL changes		
PD (mm)	Δ (0-1month)	Δ (0–3 months)
Group 1	1.63 ± 0.85	1.90 ± 0.95
	2.00 (0.00–4.00)a	2.00 (-1.00-4.00)a
Group 2	1.10 ± 0.88	1.22 ± 1.03
	1.00 (-2.00-4.00)b	1.00 (-3.00-4.00)b
Group 3	1.92 ± 0.92	2.14 ± 0.87
	2.00 (0.00–4.00)c	2.00 (0.00–4.00)c
Group 4	1.40 ± 0.95	1.51 ± 1.02
	1.00 (-1.00-4.00)d	2.00 (-3.00-4.00)d
p‡ value	*p* < 0.001	*p* < 0.001

CAL (mm)		
Group 1	1.51 ± 0.91	1.76 ± 0.99
	2.00 (0.00–4.00)a	2.00 (-1.00-5.00)a
Group 2	0.95 ± 1.04	1.05 ± 1.16
	1.00 (-3.00-5.00)b	1.00 (-3.00-5.00)b
Group 3	1.82 ± 1.02	2.04 ± 0.97
	2.00 (-1.00-5.00)c	2.00 (-1.00-5.00)c
Group 4	1.31 ± 0.99	1.42 ± 1.03
	1.00 (-1.00-4.00)d	1.00 (-3.00-4.00)d
p§ value	*p* < 0.001	*p* < 0.001

P‡ value for PD changes (baseline-1 month and baseline-3 months) in intergroup analysis (Kruskal–Wallis test and Dunn’s test.). P§ value for CAL changes (baseline-1 month and baseline-3 months) in intergroup analysis (Kruskal–Wallis test and Dunn’s test). Different letters indicate statistically significant difference for intergroup analyses

PD and CAL changes for moderately deep pockets (≥ 7 mm) were shown in Table 4. PD reduction was greater in group 3 compared to other groups between baseline and 1 month and, between baseline and 3 months (*p* < 0.05). PD reduction was greater in group 1 and group 4 compared to group 2 (*p* < 0.05), and similar in group 1 and group 4 between baseline and 1 month and, between baseline and 3 months (*p* > 0.05). CAL reduction was greater in group 3 compared to other groups between baseline and 1 month and, between baseline and 3 months (*p* < 0.05). CAL reduction was greater in group 1 and group 4 compared to group 2 (*p* < 0.05), and similar in group 1 and group 4 between baseline and 1 month and, between baseline and 3 months (*p* > 0.05).

**Table 4:** PD and CAL changes (Mean ± SD/Median (Min.-Max.) For deep pockets (PD ≥ 7 mm)

Deep pockets (≥ 7 mm) PD and CAL changes		
PD (mm)	Δ (0-1month)	Δ (0–3 months)
Group 1	2.30 ± 1.30	2.85 ± 1.52
	2.00 (-1.00-4.00)a	3.00 (0.00–7.00)a
Group 2	1.42 ± 0.77	1.45 ± 0.99
	2.00 (-1.00-2.00)b	2.00 (-1.00-3.00)b
Group 3	2.90 ± 0.87	3.39 ± 0.88
	3.00 (1.00–6.00)c	3.00 (2.00–6.00)c
Group 4	2.35 ± 1.23	2.54 ± 1.38
	2.00 (0.00–4.00)a	3.00 (0.00–5.00)a
p‡ value	*p* < 0.001	*p* < 0.001

CAL (mm)		
Group 1	1.97 ± 1.24	2.49 ± 1.51
	2.00 (-1.00-5.00)a	2.00 (-1.00-6.00)a
Group 2	1.32 ± 0.92	1.34 ± 1.09
	1.00 (-1.00-3.00)b	1.00 (-2.00-3.00)b
Group 3	2.86 ± 0.88	3.23 ± 0.93
	3.00 (1.00–6.00)c	3.00 (1.00–6.00)c
Group 4	2.29 ± 1.30	2.46 ± 1.45
	2.00 (0.00–4.00)a	3.00 (0.00–5.00)a
p§ value	*p* < 0.001	*p* < 0.001

P‡ value for PD changes (baseline-1 month and baseline-3 months) in intergroup analysis (Kruskal–Wallis test and Dunn’s test). P§ value for CAL changes (baseline-1 month and baseline-3 months) in intergroup analysis (Kruskal–Wallis test and Dunn’s test). Different letters indicate statistically significant difference for intergroup analyses

Multiple regression analysis showed that smoking was negatively associated with improvements in PD and CAL changes at baseline and 3 months post-treatment for moderately deep pockets and deep pockets, whereas omega-3 use was positively associated (Table 5). However, age and gender were not significantly associated with PD and CAL changes at baseline and 3 months post-treatment for moderately deep pockets and deep pockets (Table 5).

**Table 5:** Multiple regression analysis with PD and CAL changes (baseline-3months) for moderately deep pockets and deep pockets as dependent variable

Moderately Deep Pockets (4–6 mm)			
PD Δ(0–3)	Estimate	SE	p value
Intercept	1.405	0.460	0.003
Smoking	-0.626	0.067	<0.001
Omega-3	0.259	0.069	<0.001
Age	-0.001	0.004	0.871
Gender	-0.085	0.066	0.201
CAL Δ(0–3)			
Intercept	1.286	0.505	0.013
Smoking	-0.634	0.074	<0.001
Omega-3	0.325	0.076	<0.001
Age	-0.001	0.004	0.957
Gender	-0.064	0.073	0.381

Deep Pockets (7 mm≤)			
PD Δ(0–3)	Estimate	SE	p value
Intercept	2.945	1.331	0.030
Smoking	-1.080	0.194	<0.001
Omega-3	0.820	0.199	<0.001
Age	-0.007	0.011	0.514
Gender	0.035	0.191	0.856
CAL Δ(0–3)			
Intercept	2.666	1.398	0.061
Smoking	-0.923	0.204	<0.001
Omega-3	0.944	0.210	<0.001
Age	-0.010	0.011	0.379
Gender	0.194	0.201	0.337

## Discussion

In this retrospective study, the impact of omega-3 fatty acid supplementation as a complement to non-surgical periodontal treatment on clinical periodontal parameters was evaluated in both smokers and non-smokers with periodontitis. The results of this study demonstrated the positive impacts of using omega-3 fatty acids in addition to SI for both smokers and non-smokers with periodontitis. Another important finding of the study was that omega-3 supplementation in smokers (group 4) compensated for the negative effects of smoking, providing a healing response in deep pockets similar to that in non-smokers (group 1).

Before discussing the treatment results of the present study, it was noteworthy that baseline GI and BOP values in the smoker group (group 2) were higher than in the non-smoker group. Although it is well documented in the literature that bleeding is lower in smokers compared to non-smokers, this study had a contrary finding. This may be related to the high PI values in the smoker group. It was reported that bleeding tendency was slightly increased in plaque-covered sites in smokers [35]. In their study, Müller et al. clinically followed 65 smoking and non-smoking patients for 6 months and reported that, at the beginning of the study and at the 6th month, smokers had significantly more supra-gingival plaque compared to non-smokers. They also stated that bleeding upon probing as well as calculus were more prevalent in smokers [36]. Less bleeding in smokers is explained by the vasoconstrictor effect of nicotine [37, 38]. However, nicotine content in cigarettes varies. It is more in regular cigarettes and less in light cigarettes. In present study, the brands of cigarettes smoked by the patients and their nicotine content were not recorded, but the nicotine content of the cigarettes smoked by the individuals in the smoking group may have been low and may not have had a significant effect on gingival bleeding.

Omega-3 has been used as an adjunct to non-surgical periodontal treatment as a host modulation agent because it suppresses inflammatory eicosanoids, cytokines, MMPs and reactive oxygen species [15, 20, 28, 39]. In the present study, postoperative GI and BOP (%) levels were considerably lower in all groups compared to baseline, which are common clinical indicators showing the inflammatory progression of periodontitis. Considering the non-smoking groups, GI and BOP parameters were lower in group 3 than in group 1, but this was not statistically significant. The effectiveness of SI and oral hygiene practices in reducing symptoms of periodontal inflammation is well known. It may be thought that the possible effectiveness of omega-3 on inflammation parameters in the non-smoking group was masked by SI and oral hygiene practice, which are the gold standard in reducing inflammation. In studies that gave omega-3 in combination with low-dose aspirin and at higher doses than the dose we used in our study, it has been shown that these inflammatory parameters are significantly reduced in the groups using omega-3 compared to the control group [19, 22, 25, 28]. On the other hand, one study using a lower dose than the dose used in this study found a significant difference in inflammatory parameters in the omega-3 group compared to the control group [21], while others did not [40, 41]. In addition to all these, the severity of periodontitis of the patients in the studies, the experience of the clinician, the instruments used, the accessibility of the treated areas and the patient’s plaque control level may affect the results. No studies were found in which omega-3 was given in addition to non-surgical periodontal treatment in smokers with periodontitis. Considering the smoking groups, GI and BOP parameters were significantly lower in group 4 than in group 2. In smokers, compared to non-smokers, an increase in destructive enzymes, reactive oxygen species, cytokines such as PGE2, TNF-α, and periodontal pathogens, and a decreased healing response to periodontal treatment have been observed [42]. It was thought that the significant decrease in these inflammatory parameters in the omega-3 group of smokers compared to the control group can be explained by the capacity of omega-3 to eliminate the above-mentioned negative effects of smoking on the periodontium and healing response. SI and oral hygiene practices, which are the gold standard in resolving inflammation, may have limited effectiveness in smokers. In this study, omega-3 use in smokers may have removed or reduced this limitation and provide significant improvements in GI and BOP (%) compared to control group.

Considering recent systematic reviews and meta-analyses, omega-3 given in addition to non-surgical periodontal treatment has been shown to have a beneficial effect, especially on PD and CAL parameters [30–34]. Although whole mouth PD and CAL data were presented in this study, the main aim was to analyze the healing effectiveness of omega-3 on PD and CAL parameters in treated periodontal pockets for both smokers and non-smokers. Therefore, periodontal pockets were examined in two classes: moderately deep pockets and deep pockets. Changes in PD and CAL were examined between baseline and 1 month, as well as between baseline and 3 months. It was observed that the reductions in PD and CAL in both the moderately deep pockets and deep pockets between the baseline and 1 month and between the baseline and 3 months were significantly higher in the groups receiving omega-3 supplements than in the control groups. These results were the same in both non-smokers and smokers. These results were consistent with the results of clinical studies showing significant improvements in PD and CAL parameters in groups using omega-3 compared to the control group [19, 21, 25, 27]. As opposed to this, other investigations revealed that utilizing omega-3 PUFA during non-surgical periodontal treatment had no impact on clinical indicators [40, 41]. These variations may be caused by the use of substantially lower amounts of omega-3 PUFA than those described in the literature and in our investigation. The anti-inflammatory effect of omega-3 by reducing proinflammatory cytokines such as IL-1, IL-8, TNF-alpha, enzymes such as MMP-2, MMP-8, and MMP-9, osteoclast differentiation and activation factor RANKL, and its antioxidant properties may be related to these improvements in PD and CAL [15, 20, 23, 25, 28]. In addition, omega-3 may have contributed to this healing response by suppressing the activity and reducing the numbers of bacteria such as *Phorphyromonas gingivalis*, *Tanarella forsythia*, *Treponema denticola* and *Aggregatibacter actinomycetemcomitans*, *Fusobacterium nucleatum*, and *Prevotella intermedia* [18, 19]. Considering the healing of periodontal tissues, after non-surgical periodontal treatment, most of the healing occurs in the form of long junctional epithelium. Resolvin E1 produced by EPA has been reported to contribute to epithelial wound healing by increasing the proliferation and migration of intestinal epithelial cells [43]. Given this study, omega-3 may have such possible effects on gingival epithelial cells. When the literature on the effect of omega-3 on cells in the periodontium was reviewed, Zarrough et al. demonstrated that periodontal ligament fibroblasts were influenced by the DHA-produced resolvin D1 in a pro-wound healing, proliferative, and anti-inflammatory manner that encourages periodontal regeneration [44]. This possible positive effect of omega-3 on cells in the periodontium may be another reason that could explain the significant PD and CAL improvements in periodontal pockets. In present study, the results of multiple regression analysis also supported the significant positive effect of omega-3 on these parameters.

An interesting finding in the study was that PD and CAL changes in deep pockets between baseline and month 1, and between baseline and month 3 were statistically similar in group 1 and group 4. As the pocket depth increases, the accessibility of the instruments to that area decreases. This is a known limitation of mechanical debridement. Smoking further impedes healing response in this condition. The reason why the healing in smokers who received omega-3 supplementation was similar to that in non-smokers who did not receive omega-3 supplementation may be explained by the aforementioned anti-inflammatory and antimicrobial effects of omega-3 that compensate for this situation. Additionally, it was also demonstrated that omega-3 can decrease superoxide and hydrogen peroxide generation, inhibit MMP-2 and MMP-9, and reduce alveolar bone resorption [10]. Beneficial pro-wound healing and proliferative properties of resolving D1 on periodontal regeneration may be another reason [44]. The increase in fibronectin, which plays an important role in periodontal healing by increasing fibroblast attachment and proliferation, with omega-3 supplementation may be another factor that increases the healing response in periodontal pockets for smokers [45]. In present study, the significant positive effect of omega-3 on PD and CAL improvements was also shown by multiple regression analysis.

One of the discussion topics for this study was the differences in disease severity and progression rates among periodontitis patients in the study groups. The study population consisted of periodontitis patients with stage III and IV in terms of disease severity and grade B and C in terms of disease progression rate. When the periodontology literature was reviewed to understand whether this condition affects the healing response to non-surgical periodontal treatment, Görgülü et al. reported that improvements in PI, GI, BOP parameters and, changes in PD and CAL were similar in stage III grade B and stage III grade C groups after treatment [46]. In another clinical trial, it was demonstrated that there was no significant difference between stage III grade B and stage III grade C groups in terms of full mouth plaque and bleeding scores, PD and CAL parameters after treatment [47]. Considering these studies, it is seen that there is no difference between grade B and grade C patients in terms of response to treatment.

Various doses of omega-3 have been used in clinical studies to date. The American Heart Association has suggested that a dose of 0.5–1.8 g/day of EPA + DHA is generally regarded as safe in healthy people. Hence, a dosage of 1320 mg/day was used in this study, which was well within the range of safety dose. In some studies, it has been reported that patients complained of nausea, abdominal upsets and mouth malodor (halitosis/fish smell) as side effects [19, 22, 25]. No side effect was reported by patients in the present study.

The fact that the study groups were similar in terms of age and gender was a good situation for standardization. Primary limitation of this study is that it was conducted retrospectively. Patients in the study groups were treated by different clinicians and examiners. However, these clinicians and examiners were trained in the same periodontology clinic and followed the same treatment protocol. They were also followed by the same consultant (MS). Another limitation of the study was the short follow-up period. A third limitation is that the control groups did not use placebo drugs because the study was retrospective in design. This may have created a possible Hawthorne effect in omega-3 users. Therefore, they were likely to be more positively affected by treatment.

## Conclusions

Even with the study’s limitations, the addition of omega-3 polyunsaturated fatty acids to non-surgical periodontitis treatment produced short-term clinical advantages, especially in PD and CAL reductions for moderately deep pockets and deep pockets in both smokers and non-smokers with severe periodontitis. Patients responded well and safely to receiving omega-3 PUFAs for three months. The use of omega-3 PUFAs as an additional therapy option to treat periodontitis requires further randomized controlled trials with a larger sample size and longer duration of the follow-up.

## Data Availability

No datasets were generated or analysed during the current study.
